# Evaluation of Pain Management after Surgery: An Observational Study

**DOI:** 10.3390/medicina56020065

**Published:** 2020-02-07

**Authors:** Regina Sierżantowicz, Jolanta Lewko, Dorota Bitiucka, Karolina Lewko, Bianka Misiak, Jerzy Robert Ładny

**Affiliations:** 1 Department of Surgical Nursing, Medical University of Bialystok; Szpitalna 37, 15-295 Bialytsok, Poland; renatasierz@wp.pl (R.S.); dorota.bitucka@gmail.com (D.B.); 2 Department of Integrated Medical Care, Medical University of Bialystok, M. Skłodowskiej-Curie str. 7a, 15-096, Bialystok, Poland; 3 International Medical Students Association-Poland (IFMSA-Poland), Medical University of Białystok, Kilińskiego 1, 15-089 Bialystok, Poland; karolinka6694@gmail.com; 4 University of Medical Science, Krakowska 9,15-875 Bialystok, Poland; bianka.misiak@o2.pl; 5 Department of General and Endocrine Surgery, Medical University of Bialystok; M. Skłodowskiej-Curie 24a, 15-276 Bialystok, Poland; ladnyjr@wp.pl

**Keywords:** surgery, pain, postoperative management

## Abstract

*Background and Objectives:* Choosing a pain management strategy is essential for improving recovery after surgery. Effective pain management reduces the stress response, facilitates mobilization, and improves the quality of the postoperative period. The aim of the study was to assess the effectiveness of pain management in patients after surgery. *Materials and Methods:* The study included 216 patients operated on in the following surgical wards: the Department of Cardiosurgery and the Department of General and Endocrine Surgery. Patients were hospitalized on average for 6 ± 4.5 days. Patients were randomly selected for the study using a questionnaire technique with a numerical rating scale. *Results:* Immediately after surgery, pre-emptive analgesia, multimodal analgesia, and analgosedation were used significantly more frequently than other methods (*p* < 0.001). In the subsequent postoperative days, the method of administering drugs on demand was used most often. Patients with confirmed complications during postoperative wound healing required significantly more frequent use of drugs from Steps 2 and 3 of the World Health Organization (WHO) analgesic ladder compared with patients without complications. *Conclusion:* The mode of patient admission for surgery significantly affected the level of pain perception. Different pain management methods were used and not every method was effective.

## 1. Introduction

An important element of postoperative management is the reduction of pain caused by intraoperative tissue and/or organ damage. The intensity of pain is significantly affected by the extent of the surgical procedure, its location, and the postoperative day of monitoring, among others [1]. Choosing a pain management strategy is essential for improving recovery after surgery. The development of medicine has allowed the introduction of so-called multimodal analgesia, the aim of which is to increase the effect of the analgesics and/or suppress the severity of the nociception processes. This effect can be achieved by combining pharmaceuticals with different mechanisms of action. The most commonly used drugs in the treatment of postoperative pain included nonsteroidal anti-inflammatory drugs (NSAIDs) as well as opioids, in combination with various methods of local anesthesia, e.g., infiltration and spinal methods [1,2,3]. In postoperative pharmacotherapy, various methods of drug administration are used, e.g., doses at the patient’s request, continuous opioid infusion, patient-controlled analgesia (PCA), epidural, peripheral blocks, preventive analgesia, and analgosedation [4,5]. Effective pain management reduces the stress response, facilitates mobilization, improves the quality of the postoperative period, and influences the resumption of oral nutrition. A high standard of postoperative pain relief can achieve the “hospital without pain” certification. The aim of this study was to assess the effectiveness of pain management in patients after surgery.

## 2. Materials and Methods

### 2.1. Design and Participants

This observational study included 216 consecutive patients who were operated on in the surgical wards of the University Clinical Hospital in Bialystok at the end of 2017 and the beginning of 2018. A group of 112 patients was selected from the Department of Cardiosurgery, where coronary artery bypass grafting (CABG), transcatheter aortic valve implantation (TAVI), ascending aortic aneurysm surgery, aortic arch aneurysm repair, minimally invasive direct coronary artery bypass (MIDCAB), and procedures performed due to congenital and acquired heart defects are performed. Patients were discharged from the department 15 ± 2.1 days after surgery, on average.

A group of 104 patients was operated on at the First Clinical Department of General and Endocrine Surgery. Cholecystectomy, sleeve gastrectomy due to morbid obesity, esophageal hiatus hernia surgery, abdominal hernia repair, thyroid and parathyroid resection, and lower limb varicose vein removal were performed. All patients were operated on using the laparoscopic method. Patients were discharged from the department an average of 6 ± 4.5 days after surgery.

The study analyzed the individual medical documentation of patients regarding the admission mode, the type of surgery performed, the state of the postoperative wound, the type of analgesics used, and the effectiveness of their action. The original questionnaire consisted of 10 closed-ended questions regarding place of residence, professional activity, and housing conditions. Additionally, in order to determine the intensity of pain and the effectiveness of analgesic treatment, a numerical rating scale (NRS) with 11 degrees of pain severity was used: from 0 to 10, where 0 meant a complete lack of pain and 10 the worst imaginable pain [6,7,8]. Pain was monitored from 0 to 10 days after surgery.

### 2.2. Procedure and Ethical Considerations

The study was performed from January to December 2017. The research conforms with Good Clinical Practice guidelines, and the followed procedures were in accordance with the Helsinki Declaration. All patients signed a consent form to participate in the study. The research was approved by the Bioethics Committee of the Medical University of Bialystok (approval date 27 January 2017, no. R-I-002/313/2017).

### 2.3. Statistical Analysis

Statistical analysis was done using the Statistica 7.0 software from StatSoft Polska. In the data analysis, we used significance tests for qualitative variables (categorized), the chi-square test, and Pearson’s correlation coefficient (Pearson’s r) (X, Y). The condition for statistical significance was a level of *p* less than 0.05.

## 3. Results

### Characteristics of the Studied Group

The study included 109 women (50.46%) and 107 men (49.54%): 52 women (46.43%) and 60 men (53.57%) at the Department of Cardiosurgery and 57 women (54.81%) and 47 men (45.19%) at the First Clinical Department of General and Endocrine Surgery. The mean age of the respondents was 50 ± 11.4 years. The city was the most frequent place of residence for 62.95% of the patients. The occupational activity of the patients varied in both departments. Among the patients operated on at the Department of Cardiosurgery, there were more people on retirement and/or disability pensions (*n* = 65, 58.4%). Postoperative pain was felt by 82.87% of patients. On the NRS scale, 9 points was indicated by 8.33% of patients, 8 points by 16.20%, 7 points by 21.76%, 5 points by 16.67%, 4 points by 12.04%, 3 points by 3.70%, and the maximum value on the NRS scale of 10 points was not indicated.

In the admission to both departments, the scheduled mode was predominant (179 patients, 85.71%). Patients who were admitted in the emergency mode had significantly higher pain severity according to the NRS scale than those hospitalized in the planned (scheduled) mode. We found that the admission mode significantly affected the level of pain perception by the patient (*p* = 0.017).

**Figure 1:**
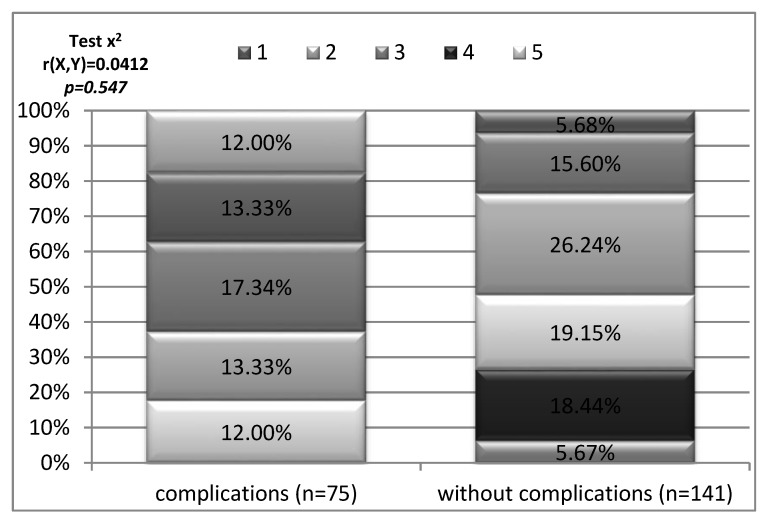
Pain intensity depending on the occurrence of complications in the healing of the postoperative wound.

The group of patients participating in the study who had postoperative wound healing complications assessed their level of pain at 7 points and above on the NRS scale. It was a higher pain rating than in the group of patients without complications in postoperative wound healing. No statistical significance was observed (Figure 1).

Low-grade pain, rated on the NRS as 1–4, was treated with nonopioid analgesics at 4–6 h intervals. Paracetamol (93.11%), ketoprofen (67.13%), and metamizole (60.19%) were most commonly used, and in individual situations, ibuprofen (4.63%) was used.

Pain of a severity of >4–6 was managed by means of a drug classified as Step 2 on the World Health Organization (WHO) analgesic ladder; in all patients it was tramadol. Administration of drugs from the first step was continued, combining tramadol with paracetamol.

In the case of painful symptoms of significant intensity (exceeding 6 on the NRS scale), which affected 46.30% of patients, drugs from the group of “strong” opioids were used. The most frequently administered were fentanyl and derivatives (84.00%), morphine (37.00%), pethidine (19.00%), buprenorphine (13.00%), or oxycodone (10.00%).

Drugs from both Steps 2 and 3 of the WHO analgesic ladder were more often administered to patients in the management of pain at the Department of Cardiosurgery (Table 1) rather than the Department of General and Endocrine Surgery.

In the analgesic treatment among the studied patients, drugs from Steps 2 and 3 of the WHO analgesic ladder were significantly more frequently administered to patients admitted in the emergency mode to both the Department of Cardiosurgery and the Department of General Surgery (Table 2) rather than in the scheduled mode.

Patients with confirmed complications during postoperative wound healing required significantly more frequent use of drugs from Steps 2 and 3 of the WHO analgesic ladder (Table 3) than patients without complications.

Immediately after surgery, the methods of pre-emptive analgesia, multimodal analgesia, and analgosedation were used significantly more frequently (*p* < 0.001) than other methods. In the subsequent postoperative days, the method of administering drugs on demand was used most often (Table 4).

Among the patients studied in the Department of Cardiosurgery, pre-emptive analgesia, multimodal analgesia, and analgosedation were used most frequently. At the Department of General and Endocrine Surgery, the administration of drugs in single doses (at the patient’s request) was adopted (Table 5).

**Figure 2:**
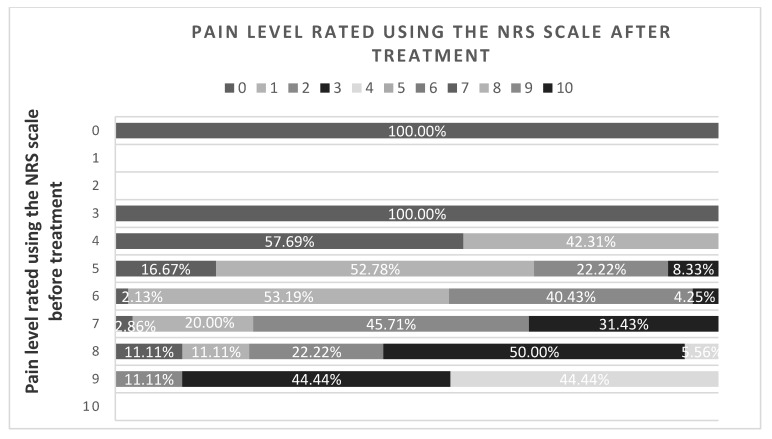
Pain assessment by the studied patients before and after pain management. Test χ2, r(X,Y) = 0.7488, *p* = 0.0001.

The applied methods of analgesic treatment used in the departments studied were effective, significantly reducing the level of perceived pain (Test χ^2^, r(X,Y) = 0.7488, *p* = 0.0001). All patients assessing the level of pain before treatment from 0 to 2 rated it at 0 on the NRS scale after treatment; among those assessing the pain level at 3, 57.69% indicated 0 and the remaining patients indicated 1 after treatment. In the group of people experiencing pain at 5 and 6 points before receiving medication, more than 90% indicated pain below 3 on the NRS after treatment. Patients evaluating pain at 9 points indicated a maximum of 4 (44.44%) after treatment (Figure 2).

## 4. Discussion

Postoperative pain management significantly reduces the number of complications associated with surgery, the time and costs of hospitalization, especially for high-risk patients (ASA scale III-IV) and patients undergoing extensive surgery. However, many studies indicate that the implemented procedures often remain inadequate for the pain intensity. This increases the risk of persistent postoperative pain and mood disorders, hinders rehabilitation, and extends the time it takes to return to full physical activity [2,8,9,10].

Pain occurs after intraoperative anesthesia subsidence, and its greatest intensity is observed on the first and second postoperative days. Systematic monitoring of pain at comparable time intervals is important, basic, and necessary. This allows the assessment of changes in the severity of pain over time. A study conducted in Denmark by Mathiesen et al. [11] among 121 patients in surgery departments indicated that 55% of patients did not assess pain intensity on any scale on the first day, 71% on the second day, and 84% on the third day. Kołodziej W. et al. [12] conducted an analysis of pain assessment in patients after surgery.

At the Department of Cardiosurgery, CABG (45.54%) procedures were the most frequently performed, as well as TAVI through the femoral artery (15.18%), ascending aortic aneurysm surgery (9.82%), and MIDCAB (7.14%). At the First Clinical Department of General and Endocrine Surgery, laparoscopic procedures were primarily performed: cholecystectomy (14.42%), gastric resection due to morbid obesity (14.42%), esophageal hiatus hernia surgery, and abdominal hernia repair (9.62%). The results of the study showed that on the second postoperative day about 90% of patients experienced pain between 2 and 4 points on the NRS. However, on the fourth day, a similar number of patients reported pain at a level of 1 to 3 points. The location of the pain depended on the surgical procedure performed.

The results of our study confirmed higher pain intensity in the immediate postoperative day at the Department of General Surgery. The low pain score obtained at the Department of Cardiosurgery can be explained by the analgosedation method, i.e., pharmacological block, and the continuous infusion of analgesics from Step 3 of the WHO analgesic ladder. In the remaining patients, pre-emptive analgesia, administration of drugs in a single dose, or combination therapy dominated. Pain reduction was achieved in all patients, which was at least 50% compared with the baseline. This indicates effective pain management in accordance with the recommendations of the American Pain Society [2,13]. One of the accepted rules of conduct is pharmacotherapy before surgery—preventive analgesia [2,3]. In our study, no such action was noted in the patient’s individual medical history or doctor’s indications. Pre-emptive analgesia was used to prevent pain.

In modern surgery, surgical site infections occur often. Among factors that have a significant impact on the occurrence of wound healing complications, Montewka M. et al. [14] indicated the increasingly complex operations performed on elderly people, who are burdened with many accompanying diseases; the use of various types of implants (mesh, prostheses, artificial valves, etc.); operating on immunocompromised patients; and the use of broad spectrum antibiotics, which increase micro-organism resistance to the pharmaceuticals used. In this study, postoperative wound complications were found in 34.72% of patients, which contributed to a higher pain score on the NRS.

Minkowitz H. et al. [15] compared pain management using the fentanyl iontophoretic transdermal system (ITS) and PCA with morphine. They found that a higher percentage of people in the group receiving fentanyl ITS discontinued treatment due to insufficient pain management compared with the PCA with morphine group. A similar percentage of patients from the fentanyl ITS and PCA with morphine groups withdrew from the study due to an adverse event. Lin T.F. et al. [16] analyzed the effect of a dexmedetomidine and morphine combination on intravenous patient-controlled analgesia. In the analysis, they showed that the addition of dexmedetomidine to the intravenous system of PCA with morphine resulted in better anesthesia, a significant saving of morphine, and less morphine-induced nausea. In addition, patients were deprived of additional sedation and unwanted hemodynamic disturbances.

In a study conducted by Fassoulaki A. et al. [17], the analgesic effect was analyzed after using multimodal analgesia in patients after mastectomy. They showed that analgesia reduces acute pain in patients, as well as at 6 and 8 months after surgery. Buvanendran A. et al. [18] described the combination of many analgesics in their paper, searching for more and more advanced pain management using multimodal analgesia. They described local anesthetic injection in the operated site as the most important method, as it provided the best results. Zalewska-Puchała J. et al. [19] showed that after thoracic surgery, patients evaluated their satisfaction with postoperative pain management above 6 points on a scale from 0 to 10.

In our study, the choice of the pain management method was influenced by the surgical procedure. Jakubów P. et al. [20] analyzed the analgesic treatment of patients after cardiac surgery on subsequent postoperative days. More side effects were observed after morphine administration compared with oxycodone. Infusion with sufentanil showed no analgesia advantage over other drugs. Systemic use of opioids required monitoring of consciousness (excessive sedation) and respiratory function (breath frequency, symptoms of hypoventilation, and hypoxia).

According to generally accepted recommendations [2,3,21], pain management should begin with nonopioids and NSAIDs. If this does not have a positive effect on the perceived pain, weak opioids should be combined with NSAIDs or nonopioid analgesics. As a last resort, when the remaining methods have been exhausted, as well as after surgical procedures causing widespread trauma, opioids should be used in combination with the other two groups of drugs. In the analyzed group of patients, Step 3 drugs on the WHO analgesic ladder were used in 46.30% of patients. In 53.70% of patients, cessation of treatment with strong opioids occurred. Step 2 drugs on the WHO ladder were administered during treatment to 43.52% of patients. Fentanyl (86.84%), ketoprofen (81.85%), metamizole (76.32%), nimesulide (71.05%), morphine (57.89%), and tramadol (55.26%) were the most commonly used.

At the Department of General and Endocrine Surgery, the administration of drugs in single doses (at the patient’s request) was used more often than at the Department of Cardiosurgery. This is due to less invasive surgical methods being used. During treatment at the patient’s request, paracetamol (91.80%), ketoprofen (64.48%), metamizole (57.92%), tramadol (43.17%), and fentanyl (30.05%) were primarily used; in a few cases, morphine was used (9.29%). The presented results are in accordance with global recommendations for pain management after surgery.

## 5. Conclusions

The mode of patient admission for surgery significantly affected the level of pain perception. Patients operated on in the emergency mode had significantly higher pain severity according to the NRS scale than those hospitalized in the planned mode. The state of the postoperative wound affected pain severity. The lowest pain intensity after the procedure was experienced by patients who did not have postoperative wound draining.

Different pain management methods were used, and not every method was effective. The highest effectiveness in the treatment of postoperative pain was achieved using analgosedation, PCA, and pre-emptive analgesia.

There is a need to introduce unified hospital procedures for postoperative pain management.

## Figures and Tables

**Table 1:** Drugs from the WHO analgesic ladder used in postoperative care.

Type of Drugs According to the WHO Analgesic Ladder		Department of Cardiosurgery (*n* = 112)	Department of General and Endocrine Surgery (*n* = 104)
Step 3 drugs	administered	64	36
		57.14%	34.62%
	cessation of treatment	48	68
		42.86%	65.38%
*Test* χ^2^, *r(x,y) = 0.2257, p < 0.001*			
Step 2 drugs	administered	56	38
		50.00%	36.54%
	cessation of treatment	56	66
		50.00%	63.46%
*Test* χ^2^, *r(X,Y) = 0.1357, p = 0.046*			

**Table 2:** Drugs from the WHO analgesic ladder used, depending on the mode of admission.

Type of Drugs According to the WHO Analgesic Ladder		Emergency Mode (*n* = 37)	Scheduled Mode (*n* = 179)
Step 3 drugs	administered	29	71
		78.38%	39.66%
	cessation of treatment	8	108
		21.62%	60.34%
*Test* χ^2^, *r(x,y) = 0.2925, p < 0.001*			
Step 2 drugs	administered	22	72
		59.46%	40.22%
	cessation of treatment	15	107
		40.54%	59.78%
*Test* χ^2^, *r(X,Y) = 0.1462, p = 0.032*			

**Table 3:** Drugs from the WHO analgesic ladder used in postoperative wound healing complications.

Type of Drugs According to the WHO Analgesic Ladder		Complication Occurrence (*n* = 75)	No Complications (*n* = 141)
Step 3 drugs	administered	61	39
		81.33%	27.66%
	cessation of treatment	14	102
		18.67%	72.34%
*Test* χ^2^, *r(x,y) = 0.5125, p < 0.001*			
Step 2 drugs	administered	46	48
		61.33%	34.04%
	cessation of treatment	29	93
		38.67%	65.96%
*Test* χ^2^, *r(X,Y) = 0.2621, p < 0.0001*			

**Table 4:** Choice of pain management method during the hospitalization of the studied patients.

Treatment Methods	Postoperative Day		
	0	1–5	6–10
Pre-emptive analgesia (prophylaxis of postoperative pain)	54	32	11
	76.06%	30.77%	42.31%
*Test* χ^2^, *r(X,Y) = 0.2515, p = 0.0001*			
Administration of drugs in single doses (on request)	42	100	26
	59.15%	96.15%	100.00%
*Test* χ^2^, *r(X,Y) = 0.3963, p = 0.0001*			
Patient-controlled analgesia (PCA)	1	0	0
	1.41%	0.00%	0.00%
*Test* χ^2^, *r(X,Y) = 0.0731, p = 0.285*			
Multimodal analgesia (combination pharmacotherapy)	46	31	8
	64.79%	29.81%	30.77%
*Test* χ^2^, *r(X,Y) = 0.2194, p < 0.001*			
Regional anesthesia techniques (epidural/subarachnoid block)	0	1	0
	0.00%	0.96%	0.00%
*Test* χ^2^, *r(X,Y) = 0.0047, p = 0.945*			
Analgosedation	32	6	0
	45.07%	5.77%	0.00%
*Test* χ^2^, *r(X,Y) = 0.4118, p < 0.0001*			

**Table 5:** Choice of pain management method in patients of the analyzed departments.

Treatment Methods	Department of Cardiosurgery (*n* = 112)	Department of General and Endocrine Surgery (*n* = 104)
Pre-emptive analgesia (prophylaxis of postoperative pain)	62	41
	55.36%	39.42%
*Test* χ^2^, *r(X,Y) = 0.1594, p = 0.019*		
Administration of drugs in single doses (on request)	80	103
	71.43%	99.04%
*Test* χ^2^, *r(X,Y) = 0.3834, p < 0.0001*		
PCA	1	0
	0.89%	0.00%
*Test* χ^2^, *r(X,Y) = 0.0657, p = 0.336*		
Multimodal analgesia (combination pharmacotherapy)	67	24
	59.82%	23.08%
*Test* χ^2^, *r(X,Y) = 0.3718, p < 0.0001*		
Transdermal therapeutic system (TTS)	1	0
	0.89%	0.00%
*Test* χ^2^, *r(X,Y) = 0.0657, p = 0.336*		
Regional anesthesia techniques (epidural/subarachnoid block)	0	1
	0.00%	0.96%
*Test* χ^2^, *r(X,Y) = 0.0708, p = 0.300*		
Analgosedation	38	0
	33.93%	0.00%
*Test* χ^2^, *r(X,Y) = 0.4452, p < 0.0001*		
